# Mitochondrial genome of *Acheilognathusbarbatulus* (Cypriniformes, Cyprinidae, Acheilognathinae): characterisation and phylogenetic analysis

**DOI:** 10.3897/BDJ.11.e93947

**Published:** 2023-02-21

**Authors:** Jinhui Yu, Xin Chen, Ruyao Liu, Yongtao Tang, Guoxing Nie, Chuanjiang Zhou

**Affiliations:** 1 College of Fisheries, Henan Normal University, Xinxiang, China College of Fisheries, Henan Normal University Xinxiang China; 2 College of Life sciences, Henan Normal University, Xinxiang City, China College of Life sciences, Henan Normal University Xinxiang City China

**Keywords:** *Acheilognathusbarbatulus*, mitochondrial genome, phylogenetic relationships

## Abstract

*Acheilognathusbarbatulus* is distributed in Yangtze River, Yellow River and Pearl River systems in China. Genome data can help to understand the phylogenetic relationships of *A.barbatulus*, but its complete mitochondrial genome has not been published. We determined the complete mitochondrial genome structure and characteristics of this species and constructed a comprehensive phylogenetic tree, based on mitochondrial genome data of several species of *Acheilognathus*, *Rhodeus* and *Pseudorasboraparva*. The complete length of the mitochondrial genome of *A.barbatulus* is 16726 bp. The genome is a covalently closed double-stranded circular molecule containing 13 protein-coding genes, two ribosomal RNAs, 22 transfer RNAs, a D-loop and a light strand replication initiation region. The base composition of the complete mitochondrial genome is A (29.33%) > T (27.6%) > C (26.12%) > G (16.95%), showing a strong AT preference and anti-G bias. All 13 PCGs have different degrees of codon preference, except for cytochrome c oxidase 1, which uses GTG as the start codon. All the PCGs use ATG as the start codon and the stop codon is dominated by TAG. The encoded amino acids Leu and Ser exist in two types, whereas the rest are all present as one type, except for tRNA^Ser (GCT)^, which lacks the D-arm and has an incomplete secondary structure, all other tRNAs can be folded to form a typical cloverleaf secondary structure. Based on the 13 PCG tandems, the Maximum Likelihood and Bayesian trees were constructed, based on the concatenated sequence of 13 PCGs for the genera *Acheilognathus* and *Rhodeus*, with *Pseudorasboraparva* as the outgroup. *Acheilognathusbarbatulus*, *Acheilognathustonkinensis* and Acheilognathuscf.macropterus were clustered together and the most closely related. The results of this study enrich the mitochondrial genomic data of *Acheilognathus* and provide molecular and genetic base information for species conservation, molecular identification and species evolution of Acheilognathinae.

## Introduction

Species in subfamily Acheilognathinae (Cypriniformes, Cyprinidae) mostly inhabit shallow and still water areas in rivers, lakes and reservoirs. *Acheilognathusbarbatulus* Günther belongs to subfamily Acheilognathinae within Cyprinidae; Acheilognathinae comprises approximately 72 species (Fan et al. 2020) and six valid genera, viz. *Acheilognathus* Bleeker, see Bleeker (1860), *Paratanakia* Chang (Chang et al. 2014), *Pseudorhodeus* Chang (Chang et al. 2014), *Rhodeus* Agassiz (Agassiz 1832), *Sinorhodeus* Li (Li et al. 2017) and *Tanakia* Jordan & Thompson (Arai and Akai 1988, Jordan and Thompson 1914). Except for *Rhodeusamarus* Bloch and *Rhodeuscolchicus* Bogutskaya & Komlev, which are distributed in Europe, the other species are mainly distributed in East and Southeast Asia (Berg 1949, Lelek 1987, Arai and Akai 1988, Bogutskaya and Komlev 2001). *Acheilognathus* is widely distributed in the Yangtze River, Min River, Han River and Yellow River in China. During the breeding season, the female fish has an extended oviduct and lays eggs in the gill cavity of mussels. Male sperm enters the inlet tube and gills of the mussel to complete fertilisation. The fertilised zygotes hatch and develop in the gills of mussels until they gain the ability to swim independently (Smith et al. 2000, Kottelat 2001, Reichard et al. 2010). Then, the zygotes leave the mussel body to complete their development. Given its unique reproductive mode and morphological diversity, *Acheilognathus* has attracted great interest from scientists. Researchers have attempted to construct a phylogenetic tree that is in line with the rate of species evolution through various single-gene analysis methods. However, the phylogenetic relationship and taxonomic status of Acheilognathinae have not been explained properly. Therefore, the genomic data of Acheilognathinae fishes must be supplemented to provide genomic resources with potential information for future evolutionary analysis of Acheilognathinae.

The mitochondrion possesses a separate genome (mitochondrial genome, mtDNA) and a relatively independent genetic system (Chinnery and Schon 2003). The mitochondrial genome of multicellular animals is usually a covalently closed double-stranded circular molecule. Linear DNA molecules are only present in certain cnidarians (Bridge et al. 1992). They have molecular lengths in the range of 14.8–19.9 Kb and the following features: tightly arranged genes (Gyllensten et al. 1985), no introns, simple structure, strict matrilineal inheritance (Li 2006), no recombination and high coding efficiency. Thus, linear DNA molecules are widely used in population genetics (Kuang et al. 2019), evolutionary biology (Chan et al. 2019) and phylogeography (Min-Shan et al. 2018).

The development of genomics has allowed the analysis of numerous mitochondrial genomes (mtDNA) and advancement of the study of population genetic structure, conservation biology and evolutionary genetics of fish (Moritz et al. 1987, Guo et al. 2004, Chen et al. 2011). Phylogenetic analysis of multiple genes in tandem can provide more accurate information than single-gene analysis. Given the relationship between evolution rate and time, different genes have various effective information sites and resolutions. To this end, the gene sequence is connected in series, which can increase the number of effective information sites. The evolution rate of 13 protein-coding genes (PCGs) is faster than that of nuclear genes and the evolution rate of each PCG is diverse, which is in line with species research at different levels (Brown et al. 1979). Therefore, we provide the detailed description (genome length and type, PCGs, non-coding genes and RNA features) and comparative analyses of the *A.barbatulus* mitochondrial genome.

*A.barbatulus* belongs to subfamily Acheilognathinae. This species is a small fish that lives in the Yangtze River, Yellow River, Pearl River and other river systems. It lays eggs in the gills of mussels and feeds on aquatic higher plants and algae. In previous studies, the phylogenetic relationship of *A.barbatulus* has not been unified in accordance with different classification methods. With the wide use of multi-site sequence analysis, we downloaded the existing mitochondrial genome data of *Acheilognathus* and *Rhodeus* fish species from the National Center for Biotechnology Information (NCBI). With *Pseudorasboraparva* as the outgroup, 13 PCGs were connected in series and Maximum Likelihood (ML) and Bayesian (Bayes) tree were constructed, based on the optimal nucleotide substitution model and optimal partition model, respectively. The description in this paper is expected to provide support and theoretical supports for the evolutionary development of Acheilognathinae in the future.

## Material and methods

### Sample collection and raw data generation

Samples of *A.barbatulus* were collected from Poyang Lake in northern Jiangxi Province, China. The experimental material for this study was provided by the Institute of Hydrobiology, Chinese Academy of Sciences. Specimens were stored in absolute ethanol at the College of Fisheries Henan Normal University. In the sampling process, the specific sampling location was not clearly marked, which led to the failure to obtain the latitude and longitude information of the sampling point. Samples from *A.barbatulus* were extracted using the phenol-chloroform protocol (Sambrook and Russell 2001), dissolved by the addition of 40 μl double-distilled water and stored at −20°C. The 30× genome of *A.barbatulus* was re-sequenced by Personalbio (Nanjing) using high-throughput sequencing technology to obtain the whole genome. The whole genome sequence of *A.barbatulus* was compared with the mitochondrial genome sequence of *Acheilognathustonkinensis* to determine the star and termination sites of the gene. Then, the mitochondrial genome was extracted from the whole genome data. MitoZ (https://github.com/linzhi2013/MitoZ) was used to obstain the complete mitochondrial genome (GenBank format) (Meng et al. 2019). We constructed libraries with 400 inserts and sequenced them using Next-generation sequencing and paired-end (PE) sequencing, based on Illumina NovaSeq sequencing platform. The bwa (0.7.12-r1039) mem programme was used to compare the filtered high-quality data with the reference genome and the parameters were compared, based on the default parameters of bwamem. Picard 1.107 software (http://www.psc.edu/index.php/user-resources/software/picard) was used to sort and convert the sam files to bam files. We used the “FixMateInformation” command to ensure consistency between all PE reads information. The total number of reads in this specie was 149005815 and the number of reads on the reference genome accounted for 92.01% of the total number of reads.

### Mitogenome annotation

The complete mitochondrial genome sequence was obtained by comparing it to the published mitochondrial genome of subfamily Acheilognathinae in NCBI. The position of start transfer RNA (tRNA^Phe^) was determined using the MITOS website (http://mitos2.bioinf.uni-leipzig.de/index.py) (Bernt et al. 2013). Preprocessing of each gene on the mitochondrial genome was based on the mitochondrial codon of bony fish and by checking whether the mitochondrial genome was ringed. This process yielded a gene map of the *A.barbatulus* mitochondrial genome. Mitofish (http://mitofish.aori.u-tokyo.ac.jp/annotation/input.html) was used to determine the positions of 13 PCGs, 22 tRNAs, two ribosomal RNA (rRNAs), control regions and the types and numbers of anticodons and start and stop codons. The tRNAscan-SE website (http://lowelab.ucsc.edu/tRNAscan-SE/) (Lowe and Chan 2016) was used to confirm the positions of tRNA genes and predict secondary structures. PCGs were translated exactly using MEGA7.0 (Sudhir et al. 2016). Base composition and relative synonymous codon usage (RSCU) were calculated by MEGA7.0. AT:GC skew was calculated using Perna's formula (Perna and Kocher 1995), with AT Skew = (A - T)/(A + T) and GC Skew = (G - C)/(G + C). CodonW 1.4.2 software (John et al. 1999) was used to obtain the codon adaptation index (CAI), effective codon number (ENC), GC and GC3.

### Comparative analyses

To study the phylogenetic position of *A.barbatulus*, we selected a part of the fish in the *Acheilognathus* and *Rhodeus* data provided by NCBI. Given that subfamilies Gobioninae and Acheilognathae are sister groups (Chen 2014), *Pseudorasboraparva* was used as the phylogenetic outgroup. A total of 33 complete mitochondrial genome sequences were selected. Table 1 shows the GenBank accession numbers and species names. Partitioned phylogenetic analysis allows the use of different nucleotide substitution models and corresponding parameters for different subsets of association data. This step helps to explore evolutionary models specific to each partition and reduce systematic errors to improve the accuracy of phylogenetic inference (Wang et al. 2012). One partitioned phylogenetic analysis method is based on Bayes principle (Ronquist and Huelsenbeck 2003) and the other is based on the ML partitioned analysis method (Stamatakis 2006). A total of 13 PCGs, two rRNA genes and 22 tRNA genes of each species were extracted using Phylosuite software (Zhang et al. 2019). MEGA7.0 (Sudhir et al. 2016) software was used to individually compare the extracted PCGs by ClustalW (John et al. 1999) (ATP6 = 684 bp, ATP8 = 165 bp, CYTB = 1141 bp, COX1 = 1551 bp, COX2 = 691 bp, COX3 = 785 bp, ND1 = 975 bp, ND2 = 1047 bp, ND3 = 351 bp, ND4 = 1381 bp, ND4L = 297 bp, ND5 = 1836 bp and ND6 = 522 bp).

The sequences were aligned, based on the mitochondrial genome by Phylosiute (Zhang et al. 2019) for the concatenation of PCGs to form a 13 PCG dataset. The best partitioning scheme and the corresponding optimal nucleotide substitution model were determined in accordance with to the Akaike Information Criterion (AIC) criteria using ModelFinder (Kalyaanamoorthy et al. 2017) and a ML tree was established by an edge-linked partitioning model based on IQ-TREE with 5000 ultrafast self-spreading values (Stamatakis 2006). Bayes were constructed, based on Bayesian Information Criterion (BIC) using PartitionFinder (Lanfear et al. 2017) to select the best partition and associated optimal base substitution model (Huelsenbeck 2001, Drummond and Rambaut 2007). Bayes inference phylogenies were inferred using MrBayes 3.2.6 (Ronquist and Huelsenbeck 2003) under an N/A model (two parallel runs, 2000000 generations), in which the initial 25% of sampled trees were discarded as burn-in.

## Data resources

GenBank accession number ON815031

## Results

### Genome size and organisation

**Figure 1.**
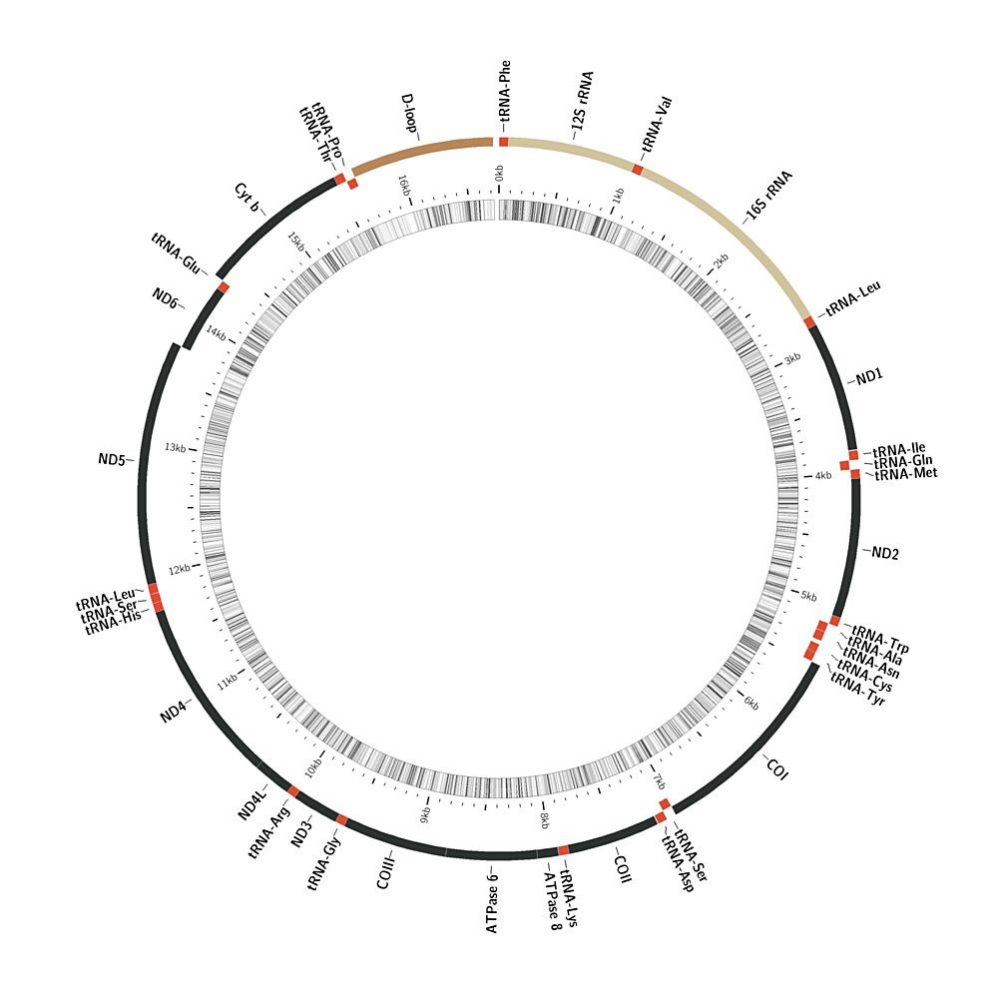
Gene map of the *Acheilognathusbarbatulus* mitochondrial genomes. The genome contained two rRNA genes (in yellow), 13 coding genes (in black), 22 tRNA genes (in red) and a control region (D-loop) (in brown). The outer ring corresponds to the H- (outermost) and L-strands and depicts the location of PCGs (except for ND6 which is encoded in the L-strand and is portrayed in red). The inner ring (black sliding window) denotes GC content along the genome.

The complete mitochondrial genome (GenBank accession number ON815031) of *A.barbatulus* is 16726 bp in total length and a typical covalently closed double-stranded cyclic molecule (Fig. 1). The mitochondrial genome of *A.barbatulus* is broadly similar to that of other vertebrates and includes 13 PCGs, namely, ATP6, ATP8, COX1, COX2, COX3, Cytb, ND1, ND2, ND3, ND4, ND4L, ND5 and ND6, 2 rRNA genes, namely, 12S rRNA and 16S rRNA genes and 22 tRNA genes, for a total of 37 genes. A non-coding region (the control region) that controls gene replication and transcription (Forst and Schulten 2001) and a light strand replication initiation region (OL) were detected. The OL is located between tRNA^Asn^ and tRNA^Cys^ and has a length of 31 bp and it can fold to form a secondary structure with a stem-loop structure. This region is highly conserved and related to the replication function of the L-strand. Except for eight tRNAs and one PCG (ND6) located on the light strand (L strand), the remaining 28 genes were located on the heavy strand (H strand) of the mitochondrial genome and the arrangement of the genes was consistent with the typical genetic composition of teleost fish (Fig. 1, Table 2).

Similar to other bony fish, spacers and overlap between genes were present. Gene overlapping promotes miniaturisation of mitotic genes, shortens genome replication time and offers a natural selection advantage (Boyce et al. 1989). The overlaps of coding genes were located between tRNA^Ile^-tRNA^Gln^, ND2-tRNA^Trp^, OL-tRNA^Cys^, ATP8-ATP6, ATP6-COX3, COX3-tRNA^Gly^, ND3-tRNA^Arg^, ND4L-ND4, ND5-ND6 and tRNA^Thr^-tRNA ^Pro^ and ten gene intervals had a total of 29 bp, which accounted for 0.17% of the total gene length. A total of 15 spacers encoding genes with a total length of 542 bp, accounting for 3.24% of the total gene length, were obtained.

### Base composition

The highest base content in *A.barbatulus* was that of A (29.33%), followed by those of T (27.6%) > C (26.12%) > G (16.95%). The complete mitochondrial genome of *A.barbatulus* showed an anti-G bias and AT preference (Table 3), a phenomenon similar to the base composition of most other teleost fishes (Perna and Kocher 1995). This base preference was also present in other parts of the mitochondrial genome, with the D-loop region, tRNAs and PCGs all having considerably greater AT content than CG content and showing a strong AT preference. Of the 13 PCGs, 10 showed an anti-A bias, except for ATP8, COX2 and ND2 which showed an A bias. Except for ND6, which showed an evident G bias, 12 genes showed anti-G bias. Meanwhile, the RNAs exhibited strong A-bias and anti-G-bias. The 12S rRNA and 16S rRNA genes with larger absolute values of AT-skew and smaller absolute values of GC-skew had a strong A-bias. ND6 and tRNAs genes have the same degree of base G-bias and T-bias. From the base composition of mitochondrial genes on both sides of *A.barbatulus*, PCGs showed a significant anti-G bias, followed by the D-loop region, 12S rRNA and 16S rRNA. Only tRNAs revealed a G bias.

### Protein-coding genes

The mitochondrial genome of *A.barbatulus* contains 13 PCGs with a total length of 11,420 bp, which accounts for 68.28% of the entire mitochondrial genome.

### Amino acid and codon usage

**Figure 2.**
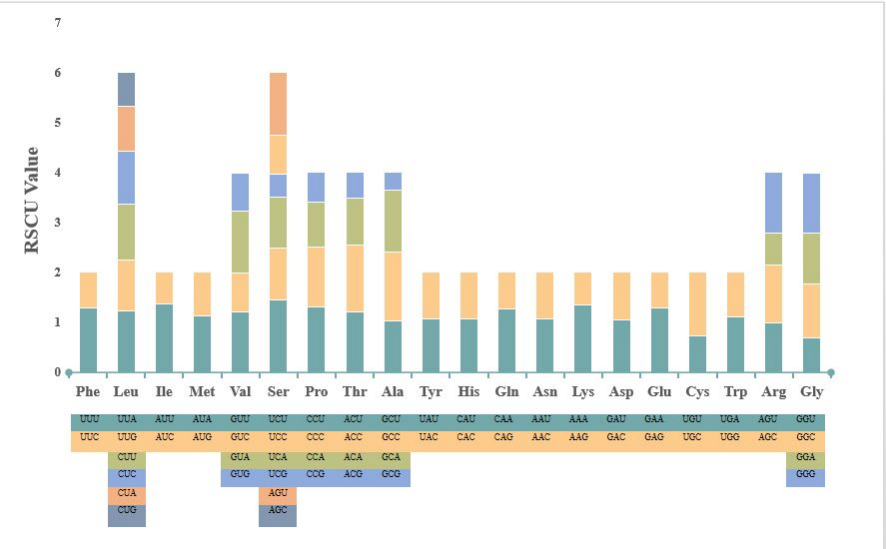
Results from analysis of Relative Synonymous Codon Usage (RSCU) of the mitochondrial genome of *Acheilognathusbarbatulus*. Codon families are plotted on the x-axis. The label for the 2, 4 or 6 codons that compose each family is shown in the boxes below the x-axis and the colours correspond to those in the stacked columns. RSCU values are shown on the y-axis.

The PCGs of *A.barbatulus* encode 3798 amino acids and, amongst the encoded amino acids, the Leu, Ser, Pro and Thr exhibit high contents. Low contents of most amino acids, such as Arg, Cys, Glu and Asp, can be observed (Table 4), with the codon encoding Leu being used the most frequently and the one encoding Cys being used the least frequently. The *A.barbatulus* codons with RSCU values > 1.0 have positive codon usage bias (CUB) and are defined as abundant codons, whereas those with RSCU values < 1.0 have negative CUB and are defined as less-abundant codons (Gun et al. 2018). The statistics of codon usage frequency and RSCU of PCGs (Fig. 2) showed that codons containing bases A and U, such as AAA, AAU and UAA, are used more frequently, whereas those containing bases C and G, such as GGG, GGC and CGC, are used less frequently. According to the comparison of RSCU, for the genes encoding *A.barbatulus* protein, the most frequently used codons were UCU (1.46%), GCC (1.38%), AAU (1.37%), and AAA (1.36%), which encode amino acids Ser, Ala, Ile and Lys, respectively, whereas codons encoding amino acids Ala (GCG, 0.36%), Ser (UCG 0.46%) and Thr (ACG, 0.52%) had the lowest codon frequency.

### Start codon and stop codon

Most of the start codons of *A.barbatulus* are ATG. Only the COX1 gene has GTG as the start codon. ND1, COX1, ATP6, ND4L, ND5 and ND6 use TAA as the complete stop codon. ND2, ATP8 and ND3 use TAG as the complete stop codon, whereas COX2, COX3, ND4 and CYTB use TA- and T-- as incomplete stop codons.

### Codon usage bias

Codon usage bias (CUB) refers to the unequal use of synonymous codons in organisms. CUB is affected by mutation and selection pressure and is an important feature of biological evolution. It not only affects gene function and expression potential, but also the accuracy and efficiency of translation. The higher the gene expression level, the stronger the CUB. To investigate codon usage preference in the mitochondrial genomes of *A.barbatulus*, we calculated CAI, ENC, GC and GC3 using CodonW1.4.2.

Effective number of condon (ENC) can describe the extent to which codon usage deviates from random selection, with values generally varying from 20 to 61. The larger the ENC value, the lower bias of expression genes towards the use of rare codons. The smaller the ENC value, the greater the preference for codons of genes with high expressions. The ENC values of PCGs in the mitochondrial genome of *A.barbatulus* ranged from 39 to 55.71 (Table 5) and were mostly concentrated in the range of 43–46, indicating a degree of codon usage preference. Codon adaptation index (CAI) values ranged from 0 to 1, with high values indicating high gene expression levels and pronounced CUB (Sharp and Li 1987). ATP8 and the three cytochrome oxidase subunits (COX1, COX2 and COX3) showed high CUB and gene expression levels. ATP6 exhibited the lowest CUB and expression levels amongst the PCGs (Table 5). GC3 refers to the GC content of the third position of all codons in a gene. In addition to methionine, tryptophan and termination codons, G and C may appear in the third codon position. The results showed that the 13 PCGs had a low GC content and a low probability of G and C in the third codon position. This finding also demonstrated that the *A.barbatulus* PCGs showed a strong AT preference (Table 3).

### tRNA and rRNA

The animal mitochondrial genome has two types of ribosomal units: the large 16S and small 12S subunits. The 16S subunit is more conserved than the 12S and the secondary structure of both rRNA genes is more conserved than the sequence (Noack et al. 1996). The 12S rRNA is located between tRNA^Phe^ and tRNA^Val^, with a total length of 956 bp, accounting for 5.72% of the complete mitochondrial genome and 50.57% of the AT content. The 16S rRNA is located between tRNA^Val^ and tRNA^Leu^, with a total length of 1660 bp, accounting for 9.92% of the total mitochondrial genome and 56.15% of the AT content.

**Figure 3.**
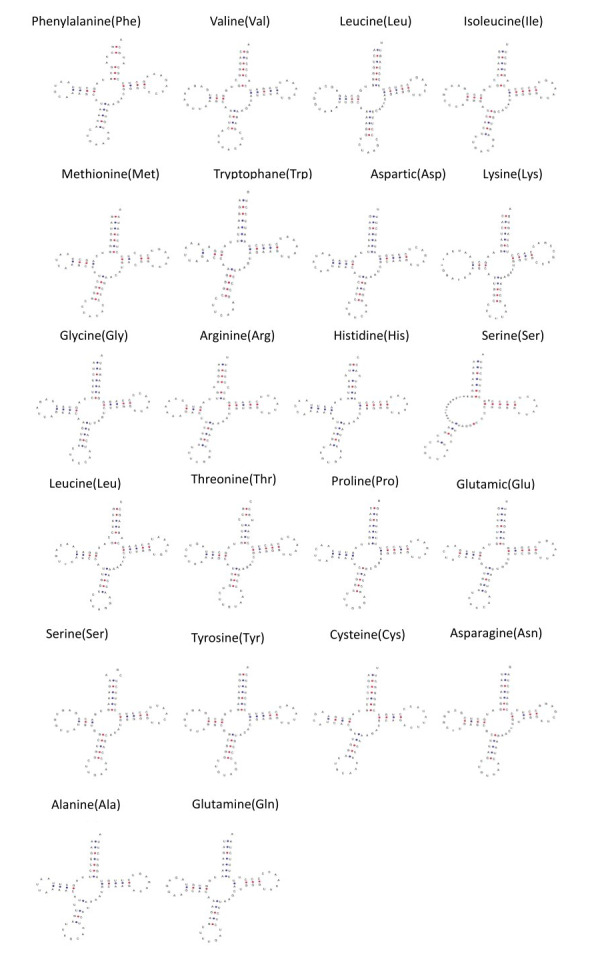
Secondary structure of the 22 tRNA genes of the mitochondrial genome of *Acheilognathusbarbatulus* predicted by tRNAScan-SE 2.0.

*A.barbatulus* has 22 tRNA genes (eight in the L-strand and 14 in the H-strand). Their individual gene lengths ranged from 68 bp to 76 bp. Except for Leu and Ser, which both contain two types, the other 18 tRNAs have only one type. Except for tRNA^Ser (GCT)^ (L strand), which lacks the D-arm resulting in an incomplete secondary structure, all 21 tRNAs can fold into the canonical cloverleaf secondary structure (Fig. 3), which is a condition that has been reported for the mitochondria of other fish species (Broughton et al. 2001, Hwang et al. 2013). The cloverleaf structure consists of four domains (AA stem and D, AC and T arms) and a variable loop (Fig. 3). For their normal functioning, these aberrant tRNAs may require co-evolutionary interaction factors or post-transcriptional RNA editing (Masta et al. 2004).

### Phylogeny and systematics of Acheilognathusbarbatulus

**Figure 4.**
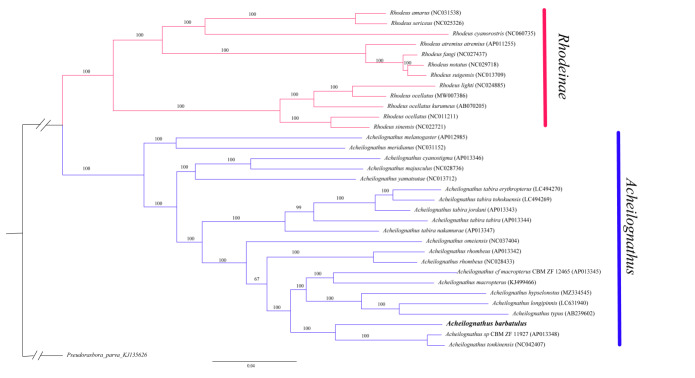
Phylogenetic trees derived from the Bayes approaches, based on concatenation of PCGs. The numbers on the nodes are the bootstrap values of Bayes. The number after the species name is the GenBank accession number. Outgroup taxa are shown, the text is bolded by the *Acheilognathusbarbatulus*.

**Figure 5.**
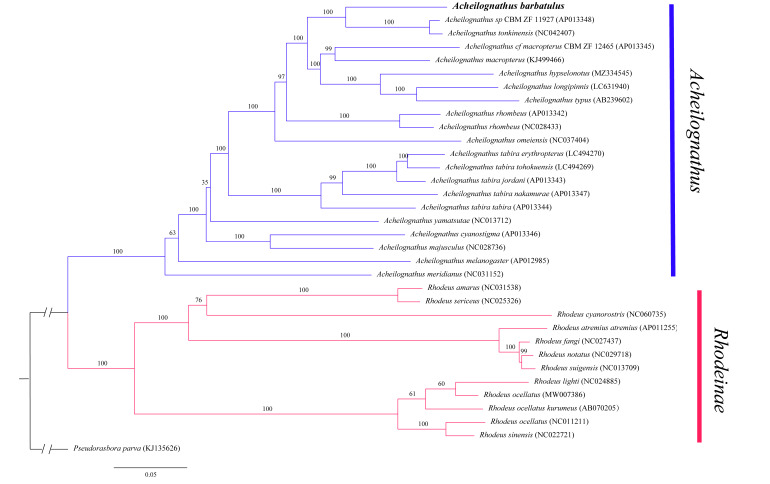
Phylogenetic trees derived from the Maximum-Likelihood (ML) approaches, based on concatenation of PCGs. The numbers on the nodes are the bootstrap values of ML. The number after the species name is the GenBank accession number. Outgroup taxa are shown, the text is bolded by the *Acheilognathusbarbatulus*.

In this study, the ML tree and MrBayes were constructed by tandemly linking 13 PCGs and using *Pseudorasboraparva* as an outgroup partition to accurately reveal the phylogenetic relationships of *A.barbatulus*. The best partitioning ML and Bayes models were based on AIC and BIC for 34 PCG tandem sequences and the most suitable nucleotide-substitution models, respectively. The best partitioning models for ML were GTR+F+I+G4: ND1, ND3, ND4, ATP6; GTR+F+I+G4: ND2; GTR+F+I+G4: ND5; GTR+F+I+G4: ND6; HKY+F+I+G4: ATP8; GTR+F+I+G4: COI; GTR+F+I+G4: COII; GTR+F+I+G4: COIII, Cytb, ND4L. The optimal partitioning models of ML and Bayesian were slightly different, with a slight difference in node support. The topological structure was consistent overall, but parts of the support rate or posterior probability of the nodes is inconsistent. This finding may be due to different ML and Bayes algorithms being different, resulting in differences in the subsequent phylogenetic trees. The results support the conclusion that *Acheilognathus* and *Rhodeus* constitute monophyletic taxa (Yang 2010) *A.barbatulus* is most closely related to *Acheilognathustonkinensis* and Acheilognathuscf.macropterus (Chen 2011). The node support rate of ML and MrBayes is 100/100 (Figs 4, 5, respectively).

## Discussion

We successfully obtained and annotated the mitochondrial genome data of *A.barbatulus*. The length of mitochondrial genome is 16726 bp, which is similar to the length of other fish genomes in Acheilognathinae, such as those of *A.signifier* (16566 bp) (Hwang et al. 2012),*A.somjinensis* (16569 bp) (Hwang et al. 2014) and *A.hypselonotus* (16706 bp) (Zhu et al. 2021). The differences in mitochondrial gene length in these species may be due to changes in the control tandem repeat sequences (Wang et al. 2020). Consistent with the absorptive stereogenomic structure of other teleost fish, the *A.barbatulus* mitochondrial genome contains 13 PCGs, two rRNA genes and 22 tRNA genes, a non-coding control region (D-loop) and OL. Genes are mainly distributed in the H strand and only ND6 and 8 tRNAs can be found in the L strand. The arrangement of genes was consistent with the typical genetic composition of Acheilognathinae (Hwang et al. 2012, Hwang et al. 2014, Zhu et al. 2021). On the basis of composition, the complete mitochondrial genome of *A.barbatulus* showed an anti-G bias and AT preference (Table 3), a phenomenon similar to the base composition of most other teleost fishes (Perna and Kocher 1995).

By analysing the relative usage frequency (RSCU) of synonymous codons of *A.barbatulus* mitochondrial genome coding genes, we observed that the RSCU values of 35 codons, such as UCU, AUU and AAA, were greater than 1 (Fig. 2), which indicates that these codons were part of the *A.barbatulus* mitochondrial genome. Amongst the 35 codons, 12 codons end with G/C and the other codons end with A/T bases (65.71%), which implies that *A.barbatulus* mitochondrial genome codons are inclined to use codons ending with A/T, whereas codons ending with G/C are less used. *A.barbatulus* mostly uses ATG and TAA as the starting codons and COX1 uses GTG as the starting codon. The termination codons are mainly incomplete codons, such as TA- and T--.

The secondary structure of *A.barbatulus* tRNA is conserved and conforms to the characteristics of fish mitochondrial genome (Broughton et al. 2001, Hwang et al. 2013). Except for tRNA^Ser^ (GCT) (L strand), which lacks the D-arm that results in an incomplete secondary structure, all 21 tRNAs can fold into the canonical cloverleaf secondary structure (Fig. 3). For normal functioning, these aberrant tRNAs may require co-evolutionary interaction factors or post-transcriptional RNA editing (Masta et al. 2004).

The mitochondrial genome of animals is maternally inherited. The nucleic acid sequence and composition are relatively conserved and the gene order is relatively stable and close. Given its structural and evolutionary characteristics, the mitochondrial genome has become an ideal object for studying the origins and evolution of animals and population genetic differentiation. Thus far, phylogenetic analysis still uses single genes as the proxy of species. However, with the continuous development of technology, the inconsistency between gene and species trees has become increasingly prominent. The information sites contained in a single gene are insufficient to reconstruct the phylogenetic relationship of a group with a gene sequence. To date, increasing number of information sites and datasets are being collected, including 13 PCGs in series, to construct phylogenetic trees.

Acheilognathinae have a complicated taxonomic history (Chang et al. 2014). Through a lot of scientific research, it is found that the phylogenetic relationship of Acheilognathinae is affected by many factors, such as different data analysis (Okazaki et al. 2001), limited in character sampling (Peilin et al. 2014) and gene sequence selection difference (Kawamura et al. 2014). As a widely distributed species of Acheilognathinae, *A.barbatulus* also has a complex taxonomic history. This study is eager to contribute to solving the taxonomic status of Acheilognathinae by expounding the phylogenetic relationship of *A.barbatulus*.

Previous studies have revealed different phylogenetic relationships in *A.barbatulus*. We first used 13 PCGs in tandem to reconstruct the phylogenetic relationship of *A.barbatulus*. Phylogenetic results showed topological differences compared with other studies due to variations in outgroups, comparative species, molecular markers and individual gene sequences (Yang 2010, Chang et al. 2014, Cheng et al. 2014, Kawamura et al. 2014, Miyake et al. 2021). Yang (2010) observed that the phylogenetic tree constructed, based on the Cyt b gene and RAG2, showed different results using various methods (Yang 2010). ML, Bayes and MJ trees of Acheilognathinae proved that *A.barbatulus* had the closest genetic relationship with *A.longibarbatus*, *A.macropterus* and *A.chankaensis*. However, the Bayes tree of Acheilognathinae constructed from RAG2 gene sequence showed that *A.barbatulus* is closely related to *A.omeiensis*, *A.tonkinensis* and *A.tabira* (Yang 2010). According to the results, the mitochondrial genes of Acheilognathinae vary greatly, the evolution rate of species is fast and the information sites represented by a single gene are limited. Therefore, single gene analysis is unsuitable for the phylogenetic study of Acheilognathinae. Kawamura K et al. (2014) used Cyt b sequences of 49 species or subspecies in three genera (*Tanakia*, *Rhodeus* and *Acheilognathus*) to construct a phylogenetic tree and observed that *A.barbatulus* is most closely related to *A.rhombens*; the authors proposed that, at the species level, the taxonomy and phylogeny between these two species are inconsistent and need to be re-assessed in future research (Kawamura et al. 2014). Based on the ND1 gene sequence, Takuya Miyake et al. (2021) concluded that *A.barbatulus* is closely related to the Japanese *A.rhombens* (Miyake et al. 2021). Chang (2014) constructed the phylogenetic relationship of Acheilognathidae, based on the tandem of six nuclear genes and Cyt b and discovered that *A.rhombens* is embedded in *A.barbatulus* and its taxonomic status has not been obtained. Thus, scientists speculated the possibility of hidden species, but further research is needed in combination with morphology (Chang et al. 2014). Cheng et al. (2014) constructed a phylogenetic tree of Acheilognathidae based on Cyt b and 12S gene sequences and obtained *A.barbatulus* and *A.longibarbatus*; meanwhile, *A.rhombens* is closely related to *A.tonkinensis* (Cheng et al. 2014).

Through the above brief description, the taxonomic status of *A.barbatulus* in Acheilognathinae has not been well solved. In this paper, the phylogenetic tree was reconstructed by tandem reconstruction of 13 PCGs in the mitochondrial genome. *A.barbatulus* is most closely related to *A.tonkinensis* and A.cf.macropterus (Chen 2014). The node support rate of ML and MrBayes is 100/100 (Figs 4, 5, respectively). Given the functional differences in various genes, these speices may have experienced different degrees of natural selection in the course of history, resulting in completely varied gene trees using different genes in molecular phylogenetic analysis. At the same time, different analytical methods may draw different conclusions when applying the mitochondrial genome to construct phylogenetic relationships. In addition, extremely rare or unrepresentative groups included in the analysis will affect the inference of final results. Compared with other studies, this paper used mitochondrial genome PCGs to build a tree in series and obtained more gene loci and a gene tree closer to the species tree. However, with the progress of technology, inconsistencies were observed between gene and species trees and these inconsistencies may be caused by incomplete pedigree sorting, hybridisation and gene flow. Additional data, such as second-generation sequencing and simplified genomes are needed to construct more accurate phylogenetic relationships.

## Conclusions

We report the mitochondrial genome sequence and characteristics of *Acheilognathusbarbatulus*. The gene structure, RNA secondary structure, D-loop region and base composition were analysed. The results contribute the mitochondrial genome data of *Acheilognathus* and provide molecular and genetic information for species conservation, molecular identification and species evolution of Acheilognathinae.

## Figures and Tables

**Table 1.** Genome sequences from NCBI in this study.

GenBank Accession	Speices	Length	AT (%)
LC494270	*Acheilognathustabiraerythropterus*	16770	56
LC494269	*Acheilognathustabiratohokuensis*	16774	56
NC028736	*Acheilognathusmajusculus*	17155	56.8
LC631940	*Acheilognathuslongipinnis*	16772	58
AP013345	Acheilognathuscf.macropterus	16761	57.6
AP013343	*Acheilognathustabirajordani*	16765	56.4
AP013342	*Acheilognathusrhombeus*	16783	56.5
AB239602	*Acheilognathustypus*	16778	57
AP013348	*Acheilognathus* sp. CBM ZF 11927	16254	56.2
AP013347	*Acheilognathustabiranakamurae*	16343	56.6
AP013346	*Acheilognathuscyanostigma*	16454	58.9
MZ334545	*Acheilognathushypselonotus*	16706	57.1
NC042407	*Acheilognathustonkinensis*	16767	56.5
NC037404	*Acheilognathusomeiensis*	16774	56.7
NC028433	*Acheilognathusrhombeus*	16780	56.9
KJ499466	*Acheilognathusmacropterus*	16773	57.6
NC013712	*Acheilognathusyamatsutae*	16703	56.7
AP012985	*Acheilognathusmelanogaster*	16556	56.8
NC031152	*Acheilognathusmeridianus*	16563	57.9
AP013344	*Acheilognathustabiratabira*	16771	56.1
NC031538	*Rhodeusamarus*	16607	55.6
AP011255	*Rhodeusatremiusatremius*	16734	55
AB070205	*Rhodeusocellatuskurumeus*	16674	56.5
NC029718	*Rhodeusnotatus*	16735	55.3
NC027437	*Rhodeusfangi*	16733	55.3
NC025326	*Rhodeussericeus*	16581	55.5
NC024885	*Rhodeuslighti*	16677	56.1
MW007386	*Rhodeusocellatus*	16574	52.9
NC013709	*Rhodeussuigensis*	16733	55.1
NC022721	*Rhodeussinensis*	16677	56.3
NC060735	*Rhodeuscyanorostris*	16580	54.9
NC011211	*Rhodeusocellatus*	16680	56.4
KJ135626	*Pseudorasboraparva*	16600	58.9

**Table 2.** Mitochondrial genes and associated features of *Acheilognathusbarbatulus*. Intergenic space (IGS) described as intergenic (+) or overlapping nucleotides.

Locus	Type	One-letter code	Strand	Amino acids	Position			Codon			
					Start	Stop	Length (bp)	Start	Stop	Anti-condon	Intergenic nucleotide
tRNA^Phe^	tRNA	F	H		1	69	69			GAA	0
12S rRNA	rRNA		H		70	1025	956				1
tRNA^Val^	tRNA	V	H		1027	1098	72			TAC	16
16S rRNA	rRNA		H		1115	2774	1660				0
tRNA^Leu^	tRNA	L2	H		2775	2850	76			TAA	0
NAD1	Protein-coding		H	324	2851	3825	975	ATG	TAA		4
tRNA^Ile^	tRNA	I	H		3830	3901	72			GAT	-2
tRNA^Gln^	tRNA	Q	L		3900	3970	71			TTG	1
tRNA^Met^	tRNA	M	H		3972	4040	69			CAT	0
NAD2	Protein-coding		H	348	4041	5087	1047	ATG	TAG		-2
tRNA^Trp^	tRNA	W	H		5086	5155	70			TCA	1
tRNA^Ala^	tRNA	A	L		5157	5225	69			TGC	1
tRNA^Asn^	tRNA	N	L		5227	5299	73			GTT	2
OL			H		5302	5332	31				-2
tRNA^Cys^	tRNA	C	L		5331	5398	68			GCA	0
tRNA^Tyr^	tRNA	Y	L		5399	5469	71			GTA	1
COX1	Protein-coding		H	516	5471	7021	1551	GTG	TAA		0
tRNA^Ser^	tRNA	S2	L		7022	7092	71			TGA	2
tRNA^Asp^	tRNA	D	H		7095	7165	71			GTC	7
COX2	Protein-coding		H	230	7173	7863	691	ATG	T(AA)		0
tRNA^Lys^	tRNA	K	H		7864	7939	76			TTT	1
ATP8	Protein-coding		H	54	7941	8105	165	ATG	TAG		-7
ATP6	Protein-coding		H	227	8099	8782	684	ATG	TAA		-1
COX3	Protein-coding		H	261	8782	9566	785	ATG	TA(A)		-1
tRNA^Gly^	tRNA	G	H		9566	9636	71			TCC	0
NAD3	Protein-coding		H	116	9637	9987	351	ATG	TAG		-2
tRNA^Arg^	tRNA	R	H		9986	10054	69			TCG	0
NAD4L	Protein-coding		H	98	10055	10351	297	ATG	TAA		-7
NAD4	Protein-coding		H	460	10345	11725	1381	ATG	T(AA)		0
tRNA^His^	tRNA	H	H		11726	11794	69			GTG	0
tRNA^Ser^	tRNA	S1	H		11795	11863	69			GCT	1
tRNA^Leu^	tRNA	LI	H		11865	11937	73			TAG	0
NAD5	Protein-coding		H	611	11938	13773	1836	ATG	TAA		-4
NAD6	Protein-coding		L	173	13770	14291	522	ATG	TAA		0
tRNA^Glu^	tRNA	E	L		14292	14360	69			TTC	2
CYTB	Protein-coding		H	380	14363	15503	1141	ATG	T(AA)		0
tRNA^Thr^	tRNA	T	H		15504	15576	73			TGT	-1
tRNA^Pro^	tRNA	P	L		15576	15645	70			TGG	406
D-loop	Non- coding		H		16052	16630	579				96

**Table 3.** Nucleotide composition of the complete *Acheilognathusbarbatulus* mitochondrial genomes (and concatenated PCGs, rRNA, D-loop) analysed in this study.

Region		Base composition (%)						
	Total	T	C	A	G	AT（%）	ATskew	GCskew
ATPase 6	683	30.75	28.11	26.35	14.79	57.10	-0.08	-0.31
ATPase 8	165	27.27	24.24	34.55	13.94	61.82	0.12	-0.27
COI	1551	30.82	26.05	25.02	18.12	55.83	-0.10	-0.18
COII	691	29.09	25.47	29.52	15.92	58.61	0.01	-0.23
COIII	784	29.97	26.28	25.13	18.62	55.10	-0.09	-0.17
Cyt b	1141	31.11	26.12	27.70	15.07	58.81	-0.06	-0.27
ND1	975	30.67	27.49	25.74	16.10	56.41	-0.09	-0.26
ND2	1045	26.22	31.39	27.75	14.64	53.97	0.03	-0.36
ND3	349	30.37	28.08	25.50	16.05	55.87	-0.09	-0.27
ND4	1381	29.76	27.23	28.39	14.63	58.15	-0.02	-0.30
ND4L	297	29.29	28.28	26.94	15.49	56.23	-0.04	-0.29
ND5	1836	29.74	26.96	28.70	14.60	58.44	-0.02	-0.30
ND6	522	37.93	13.60	15.52	32.95	53.45	-0.42	0.42
PCGs	11420	30.17	26.58	26.73	16.52	56.89	-0.06	-0.23
tRNAs	1561	27.35	21.33	28.76	22.55	56.12	0.03	0.03
D-loop	579	29.53	24.01	28.32	18.13	57.86	-0.02	-0.14
12S rRNA	957	19.54	26.96	31.03	22.47	50.57	0.23	-0.09
16S rRNA	1676	21.30	22.49	34.84	21.36	56.15	0.24	-0.03
Complete genome	16726	27.60	26.12	29.33	16.95	56.93	0.03	-0.21

**Table 4.** Results from the Relative Synonymous Codon Usage (RSCU) analysis for the PCGs of the mitochondrial genome of *Acheilognathusbarbatulus*. * denotes stop codon.

AA	Codon	Count	RSCU	AA	Codon	Count	RSCU
Phe	UUU(F)	150	1.3	Tyr	UAU(Y)	145	1.08
	UUC(F)	81	0.7		UAC(Y)	123	0.92
Leu	UUA(L)	127	1.24		UAA(*)	155	1.53
	UUG(L)	104	1.01		UAG(*)	96	0.95
	CUU(L)	114	1.11	His	CAU(H)	121	1.08
	CUC(L)	109	1.06		CAC(H)	103	0.92
	CUA(L)	92	0.9	Gln	CAA(Q)	125	1.28
	CUG(L)	70	0.68		CAG(Q)	70	0.72
Ile	AUU(I)	151	1.37	Asn	AAU(N)	160	1.08
	AUC(I)	69	0.63		AAC(N)	135	0.92
Met	AUA(M)	107	1.14	Lys	AAA(K)	153	1.36
	AUG(M)	81	0.86		AAG(K)	72	0.64
Val	GUU(V)	47	1.21	Asp	GAU(D)	71	1.06
	GUC(V)	30	0.77		GAC(D)	63	0.94
	GUA(V)	48	1.24	Glu	GAA(E)	96	1.29
	GUG(V)	30	0.77		GAG(E)	53	0.71
Ser	UCU(S)	128	1.46	Cys	UGU(C)	42	0.74
	UCC(S)	90	1.03		UGC(C)	72	1.26
	UCA(S)	88	1.01	Trp	UGA(W)	84	1.11
	UCG(S)	40	0.46		UGG(W)	67	0.89
Pro	CCU(P)	166	1.32	Arg	CGU(R)	39	1
	CCC(P)	149	1.18		CGC(R)	45	1.15
	CCA(P)	113	0.9		CGA(R)	25	0.64
	CCG(P)	75	0.6		CGG(R)	47	1.21
Thr	ACU(T)	121	1.21	Ser	AGU(S)	68	0.78
	ACC(T)	133	1.33		AGC(S)	111	1.27
	ACA(T)	95	0.95		AGA(*)	75	0.74
	ACG(T)	52	0.52		AGG(*)	80	0.79
Ala	GCU(A)	55	1.03	Gly	GGU(G)	36	0.7
	GCC(A)	74	1.38		GGC(G)	55	1.07
	GCA(A)	66	1.23		GGA(G)	52	1.01
	GCG(A)	19	0.36		GGG(G)	62	1.21

**Table 5.** Preference for codon usage of genes encoding proteins in mitochondrial genome of *Acheilognathusbarbatulus*.

	CAI	ENC	GC	GC3
ATP6	0.117	48.05	0.432	0.361
ATP8	0.257	44.06	0.388	0.333
COI	0.177	44.77	0.446	0.374
COII	0.186	52.50	0.416	0.332
COIII	0.196	46.88	0.454	0.380
Cytb	0.160	43.52	0.415	0.363
ND1	0.121	46.39	0.440	0.361
ND2	0.139	45.89	0.464	0.399
ND3	0.126	44.61	0.445	0.400
ND4	0.125	45.72	0.422	0.343
ND4L	0.116	39.00	0.443	0.365
ND5	0.152	47.11	0.418	0.394
ND6	0.141	45.53	0.470	0.442
PCGs	-0.037	55.71	0.420	0.444
